# Anger fosters action. Fast responses in a motor task involving approach movements toward angry faces and bodies

**DOI:** 10.3389/fpsyg.2015.01240

**Published:** 2015-09-03

**Authors:** Josje M. de Valk, Jasper G. Wijnen, Mariska E. Kret

**Affiliations:** ^1^Department of Psychology, University of Amsterdam, Amsterdam, Netherlands; ^2^Amsterdam Brain and Cognition Center, Amsterdam, Netherlands; ^3^Cognitive Psychology Unit, Institute of Psychology, The Faculty of Social and Behavioural Sciences, Leiden University, Leiden, Netherlands; ^4^Leiden Institute for Brain and Cognition, Leiden, Netherlands

**Keywords:** emotion perception, body posture, facial expression, affect, reaction times, action preparedness

## Abstract

Efficiently responding to others’ emotions, especially threatening expressions such as anger and fear, can have great survival value. Previous research has shown that humans have a bias toward threatening stimuli. Most of these studies focused on facial expressions, yet emotions are expressed by the whole body, and not just by the face. Body language contains a direct action component, and activates action preparation areas in the brain more than facial expressions. Hence, biases toward threat may be larger following threatening bodily expressions as compared to facial expressions. The current study investigated reaction times of movements directed toward emotional bodies and faces. For this purpose, a new task was developed where participants were standing in front of a computer screen on which angry, fearful, and neutral faces and bodies were presented which they had to touch as quickly as possible. Results show that participants responded faster to angry than to neutral stimuli, regardless of the source (face or body). No significant difference was observed between fearful and neutral stimuli, demonstrating that the threat bias was not related to the negativity of the stimulus, but likely to the directness of the threat in relation to the observer. Whereas fearful stimuli might signal an environmental threat that requires further exploration before action, angry expressions signal a direct threat to the observer, asking for immediate action. This study provides a novel and implicit method to directly test the speed of actions toward emotions from the whole body.

## Introduction

Humans are well adapted to quickly recognize and adequately respond to another’s emotional expression. As emotional expressions are intrinsically linked to actions, it has been proposed that in our attempt to understand emotions, we should study *actions* rather than cognitions or feelings (Frijda et al., 1989). Because threatening signals are thoroughly processed (Williams et al., 2006) and rapidly detected (Hansen and Hansen, 1988; Öhman et al., 2001b; Pinkham et al., 2010), they prepare for greater action preparedness (Schutter et al., 2008; Van Loon et al., 2010; Borgomaneri et al., 2014) and for quick actions (Coombes et al., 2005). For example, fearful and angry as compared to neutral facial expressions induce larger peak amplitudes on early face-related components such as the N170 and VPP (120–220 ms post-stimulus; Williams et al., 2006; Hinojosa et al., 2015), but also boost later potentials reflecting decision making processes (Lang et al., 1990; Liddell et al., 2004). There is an extensive literature on the implicit (i.e., non-conscious) processing of facial expressions. Research showing that faces and facial expressions are still processed and yield similar actions under conditions of limited attention and awareness has contributed significantly to the view that faces have a special status (Eastwood and Smilek, 2005; Johnson, 2005; Vuilleumier, 2005; de Gelder et al., 2010, 2014).

The majority of emotion studies focus on facial expressions. However, faces are naturally encountered in the context of a whole body. Distinct expressions of emotion portrayed by body language are readily recognized even in the absence of facial and vocal cues (Bannerman et al., 2009; de Gelder et al., 2010; de Gelder and Van den Stock, 2011). Moreover, the perception of facial expressions is strongly influenced by body language and the other way around, i.e., the interpretation of a face (body) can change, depending on the emotion expressed by the body (Meeren et al., 2005; Van den Stock et al., 2007; Willis et al., 2011; Van den Stock and de Gelder, 2012, 2014; Kret et al., 2013a,b; Martinez et al., 2015). Eye-tracking studies have shown that when humans are observing whole-body images of other individuals, they generally spend more time looking at the face than at the posture (Kret et al., 2013a,b). However, when the observed individuals display conflicting messages through the face and the body (for example, a happy face above an angry body), then visual attention immediately allocates toward the threat, whether expressed by the face or by the body (Kret et al., 2013a,b). Other studies have shown that recognition of emotional bodies is facilitated (or hindered) by simultaneous presentation of task-irrelevant congruent (or incongruent) emotional facial expressions, respectively (Willis et al., 2011; Gu et al., 2013). As with facial expressions, body language can also be processed without awareness (Tamietto et al., 2009, 2015; Tamietto and de Gelder, 2010; Van den Stock et al., 2011). For example, in a study with patients with hemi-spatial neglect, fearful bodily expressions automatically summoned spatial attention toward the neglected side (Tamietto et al., 2007).

Faces and bodies are processed by similar neural networks (Van de Riet et al., 2009). However, as compared to facial expressions, bodily expressions of emotion contain a direct action component; a fearful posture is bending backward/avoiding the observer, and an aggressive posture is leaning to the front/approaching the observer. This core difference is reflected in distinct brain activity patterns. For example, it has been shown that body language activates action preparation areas more than facial expressions, especially when fear or anger is expressed, and even more so when expressed by a male versus female (Kret et al., 2011a).

Although fear and anger are both negative emotions, a fearful signal can be more ambiguous and might signal an environmental threat, whereas anger can be perceived as a direct threat toward the observer requiring immediate action (Grillon and Charney, 2011). Merely looking at fearful faces does not evoke an autonomic response (Dunsmoor et al., 2009) or subjective fear (Davis and Whalen, 2001). Rather, fearful faces are important signals for a potential threat in one’s environment, leading to increased vigilance for the source of danger without concomitant defensive mobilization (Whalen, 1998; Whalen et al., 1998). Pichon et al. (2009) directly compared brain activity during the perception of fearful and angry body expressions. They observed that angry body expressions activated the premotor cortex more than fearful expressions. In addition, Kret and de Gelder (2013) showed that angry bodies were more distracting than fearful bodies in a matching-to-sample task, slowing down reaction times when the task required an action away from the angry cue (in violent offenders and control males alike). Together, these studies suggest enhanced action preparation in response to anger than fear.

Thus far, fear and anger have never been directly compared in a task that requires direct actions toward these expressions. By using a new experimental paradigm, the current study investigates the speed of movements toward angry, fearful, and neutral facial and bodily expressions by male actors. We opted for the threat related emotions fear and anger for three reasons. First, both emotions can be expressed equally well via the body and the face, contrary to for example disgust that is not well recognized from body expressions alone, and happiness that is much better expressed by the face as compared to the body (de Gelder et al., 2010). Second, fear and anger are both negative emotions, similarly arousing and contain a clear action component in the body expression, in contrast to for example a sad body expression (Pichon et al., 2008; Kret et al., 2011a) Third, anger is a more direct threat than fear.Male actors were included exclusively because previous research has shown they evoke greater affective responses than female actors, at least for the emotions fear and anger (Kret et al., 2011b)

In real life, people seldom explicitly label other’s emotional expressions, yet this is what is most commonly asked during lab experiments. In explicit tasks such as emotion recognition tests, other cognitive processes such as memory and decision making can interfere with emotional processes such as action preparedness (for example, see Kliemann et al., 2013). In implicit tasks, this interference is much less of an issue. An extensively used implicit task is the emotional dot probe task which, because it requires minimal training, is often used in studies with children and non-human primates (King et al., 2012; Lacreuse et al., 2013; Parr et al., 2013).

In the classical dot-probe task two emotional stimuli are presented simultaneously and are followed by a dot that is presented on the left or right side of the screen. Typically, participants are sitting in a chair and required to click on a left or right button on a keyboard or button box. What is generally measured is an attentional bias score, i.e., the extent to which reaction times are shortened when the dot was presented at the location of the emotional as compared to the neutral image. The aim of the current study is not to measure competition between two different stimuli, but to measure the speed of an action toward expressions and in addition, to provide a novel tool for future research. For that reason, we designed a simplified version of the emotional dot probe task where one image is presented at a time, on either the left or on the right side of the screen. To increase the naturalness of the study, a touch screen was used, in front of which participants were standing. Participants were requested to *directly* tap on the image as fast as they could.

Our expectations were threefold. First, we expected faster reaction times following threatening as compared to neutral stimuli. Second, bodies contain a direct action component and faces do not, which is why we expected faster responses toward fearful and angry bodies than toward faces. Third, in line with research showing greater activation of motor preparation areas in the brain and greater action preparedness following angry versus fearful stimuli, we predicted shorter reaction times following angry as compared to fearful bodies (Whalen, 1998; Davis and Whalen, 2001; Pichon et al., 2009).

## Materials and Methods

### Participants

Thirty-three participants (12 male) took part in the experiment. The mean age was 23.23 (SD = 4.35), with age ranging from 18 to 35 years old. The participants were recruited at the psychology laboratory from the University of Amsterdam. They filled out an informed consent and were debriefed after the experiment for which they obtained course credit or money. Participants had no neurological or psychiatric history, were right-handed and had normal or corrected-to normal vision. The study was performed in accordance with the Declaration of Helsinki and approved by the local medical ethical committee.

### Procedure

**FIGURE 1:**
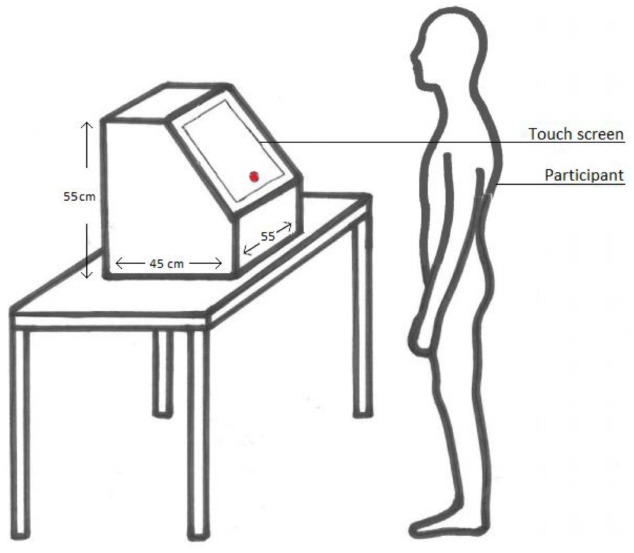
**Experimental Setup**.

After participants read the information brochure and signed the informed consent, they were given verbal instructions. In order to investigate the interference of bodily and facial expressions on the emotion task, angry, fearful, and neutral face or body expressions were randomly presented with Presentation software (Neurobehavioral Systems, San Francisco, CA, USA). Participants were asked to stand behind the touch screen (Figure 1). The distance between the participants and touch screen was 50 cm, a distance at which all participants could comfortably touch the screen. They were instructed to press the red dot that appeared on the screen to start the trial and to subsequently press the appearing image as quickly as possible.

**FIGURE 2:**
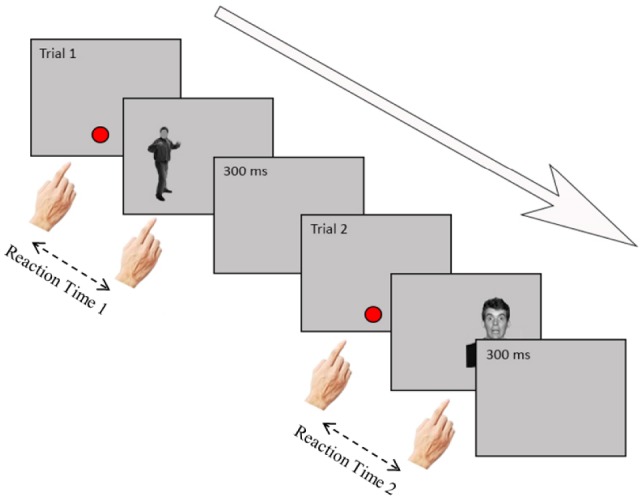
**Timeline of two trials in the touch screen task.** At the start of a trial, participants touched the red dot to make it disappear. Then, an image (angry/fearful/neutral face or body) appeared at the left or right side of the screen. Participants had to touch the image as quickly as possible. A blank screen appeared for 300 ms, after which a new trial started.

A trial started with the presentation of a dot in the middle, lower side of the screen, on which participants had to tap with their right hand. Because participants had to visually guide their hand action, this guaranteed that their eye fixation was on the location of the dot and that the hand did not occlude the upcoming stimulus. Immediately after participants touched the dot, the emotional picture was randomly presented on the left or on the right side of the screen. Participants were instructed to tap as fast as they could directly on the image, after which the image disappeared. Participants completed 192 trials which took about 5 min. Reaction times were measured from the moment the participant pressed the red dot in the center of the screen to the moment the participant pressed the image. See Figure 2 for a trial outline.

### Materials

**FIGURE 3:**
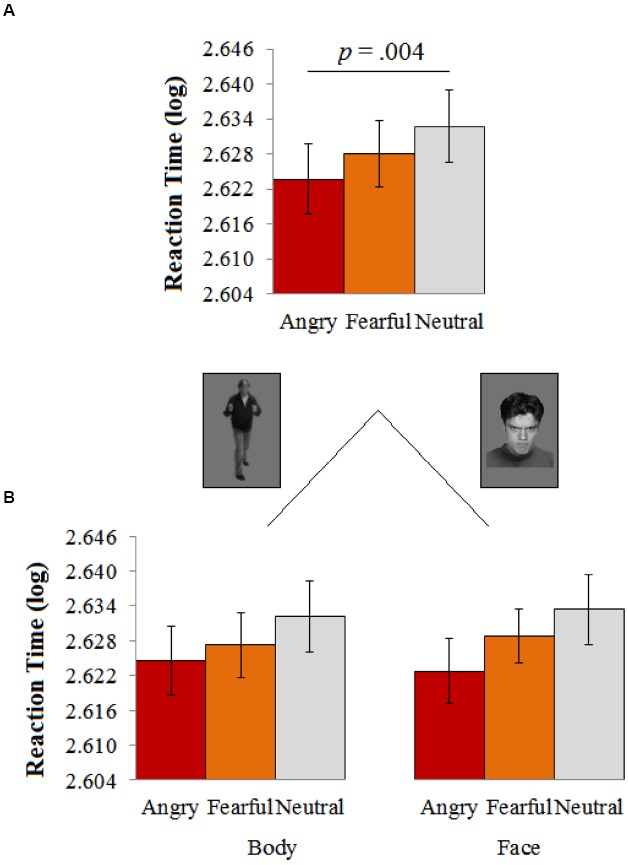
**(A)** Participants were faster in responding to angry as compared to neutral expressions. **(B)** The faster response following anger was independent of the source (face or body). Error bars represent the standard error (SE) of the mean.

Pictures consisted of angry, fearful, and neutral body postures and facial expressions. The bodies were taken from the BEAST stimulus data base (de Gelder and Van den Stock, 2011) and the faces from the NimStim set (MacArthur Research Network on Early Experience and Brain Development, 2002). All face and body stimuli were from male actors. For the body stimuli, the facial features were blurred so that the emotional signal could only be perceived from the posture. The images were turned to grayscale and had an average gray background color. The luminance of each image was set to the average of all stimuli. Bodies and faces of 16 different identities of men (32 in total) were used, each of which expressed fear or anger or which showed a neutral expression (96 trials in total). These stimuli and their mirror images were repeated twice (192 trials in total). The diameter of the dot was 2.5 cm. The size of the images of both faces and bodies was 17 cm (width) by 25 cm (height), but see the stimulus examplars in Figures 2 and 3 for the proportions.

### Experimental Design

The study had a two within subjects (source: body/face) by three within subjects (emotion: anger, fear, or neutral) design.

### Data-Analysis

The reaction time data was skewed (skewness = 12.813, SE = 0.032) and had outliers (maximum reaction time = 9930 ms, mean reaction time = 470 ms). The data was therefore filtered to exclude reaction times that were more than two standard deviations above each individuals mean reaction time. Next, a log-transformation was performed. After these steps, the issue of skewness was solved (skewness = 1.019, SE = 0.033) and inspections of the histograms and Shapiro–Wilks tests showed that each experimental condition was normally distributed (*p* > 0.064). Data were analyzed in a repeated measures ANOVA implemented in IBM SPSS Statistics 20. Significant effects were followed up by Bonferroni-corrected pairwise comparisons.

## Results

A 3 (Emotion: Angry/Fear/Neutral) × 2 (Source: Face/Body) repeated measures ANOVA showed a main effect of emotion *F*(2,64) = 7.173, *p* = 0.002, ${\text{η}}_{\text{p}}^{\text{2}}$ = 0.183^1^, 1 – β = 0.922, with faster (log-transformed) reaction times following angry (*M* = 2.624, SE = 0.005) as compared to neutral expressions (*M* = 2.633, SE = 0.006, *p* = 0.004), independent from the source [face or body: *F*(2,64) = 0.225, *p* = 0.799, ${\text{η}}_{\text{p}}^{\text{2}}$ = 0.007]. A Bonferroni-corrected pairwise comparison showed that the difference between fear (*M* = 2.628, SE = 0.005) and neutral (*M* = 2.633, SE = 0.006) was not significant (*Mean Difference* = 0.005, SE = 0.002, *p* = 0.190)^2^. A similar comparison between fear and anger yielded no significant difference either, *p* = 0.148^3^ (see Figure 3).

## Discussion

Emotions are intrinsically linked to actions (Frijda et al., 1989). The aim of the current study was to measure the speed of actions in response to emotional stimuli and to provide a new tool for measuring this, which can be implemented in future studies. In most previous studies investigating emotions and actions, participants were sitting in front of a computer screen and only indirectly responded to emotions. In the current study, participants were *standing* behind a touch screen on which pictures of fearful, angry and neutral faces and bodies appeared, which they had to tap on as fast as they could. Results showed that participants responded faster to angry as compared to neutral stimuli, regardless of whether the emotion was portrayed in the face or the body. No significant difference in response latencies was found between fearful and angry or fearful and neutral stimuli.

Many emotion studies have reported a threat bias in which threatening faces or scenes are prioritized over neutral (Hansen and Hansen, 1988; Öhman et al., 2001b; Williams et al., 2006; Pinkham et al., 2010). Our study adds to these findings by showing that angry bodies, like angry faces, trigger faster actions, supporting the idea of a general threat bias, independent of the source. Although a fearful expression is a threat cue as well, no reaction time difference was observed between fearful and neutral cues, possibly because this expression reflects a different source of threat, i.e., environmental and indirect rather than direct and personal (Whalen, 1998; Davis and Whalen, 2001; Adams et al., 2003). In other words, an angry person might be perceived as a direct threat to the participant but a fearful person might be more ambiguous as the source of the threat is unclear. This suggests that a direct threat (anger), asks for immediate action and an ambiguous threat (fear) requires exploration and hence more processing time and a somewhat slower response. It is possible that different results may be obtained if the eye gaze of the fearful stimulus is averted rather than directed at the participant. This would also be interesting to test in a cueing paradigm. In sum, the threat bias reported in this study was probably related to the directness of the threat and not to the negativity of the stimuli.

Previous emotion studies mainly focused on faces. However, emotions are displayed in both the face and the body, the main difference being that bodies contain an action component and faces do not. We hypothesized that this action component would evoke faster actions in our participants, resulting in shorter response latencies when responding to bodies than to faces. Yet, no difference between bodies and faces was observed. Two explanations are possible. First, the results could indicate that it does not matter whether threat is displayed by the body or the face. A threat is a threat and it has evolutionary benefits to be able to quickly respond to that. Another explanation can be found in the nature of the stimulus material. Faces and bodies were not presented in true proportions: the size of the pictures was identical, which means that faces were displayed relatively larger than bodies. Bigger stimuli are seen as closer in distance and the relatively large faces may have therefore overshadowed a putative difference in reaction times between faces and bodies. To rule out this explanation, a future study should therefore present faces and bodies in true proportions.

In a previous study, Bannerman et al. (2009) aimed to pull apart effects of emotional faces and bodies on the speed of actions as measured through manual responses from effects on attention as measured through eye fixations. In their study, fearful/neutral body or face pairs were bilaterally presented for either 20 or 500 ms. Results showed faster saccadic orienting to fearful body and face emotions compared with neutral only at the shortest presentation time (20 ms). For manual responses, faster discrimination of fearful bodies and faces was observed only at the longest duration (500 ms). These results suggest faster localization of threat conveyed both by the face and the body within the oculomotor system. In addition, enhanced detection of fearful body postures suggests that we can readily recognize threat-related information conveyed by body postures in the absence of any face cues. One shortcoming of the study is that whereas fixations landed directly on the stimulus, movements did not and landed on a response box instead. Importantly, this and other studies all suggest that the core function of emotion may be to coordinate attention and action preparedness and other cognitive functions, in order to facilitate adaptation to environmental challenges (Öhman et al., 2001a,b; Lewis, 2005; Pichon et al., 2008, 2009; Schutter et al., 2008; Van Loon et al., 2010).

In the current study, the mechanism underlying the faster responses toward angry as compared to neutral stimuli remains speculative and might reflect enhanced attention (Wiers et al., 2010), increased readiness to act (e.g., Schutter et al., 2008; Van Loon et al., 2010; Borgomaneri et al., 2014) or both (for a recent discussion, see Frijda et al., 2014). Future studies could make use of our procedure and aim to disentangle effects of threat on attention and the speed of an approach action readiness. One approach would for example be to have participants stand in front of the screen and present pictures on the left, on the right or in the middle of the touch screen. The dot that needs to be tapped on at the start of a trial is then always presented in the middle of the screen. A picture presented at that exact location therefore does not require a shift in attention. If the threat bias disappears for centrally presented stimuli, the threat bias found in the current study would be completely driven by an attention bias. If the threat bias stays for centrally presented stimuli, the threat bias in this study does reflect action readiness.

The task employed in the current study required an approach-related movement. It is possible that different results can be obtained using an avoidance-related movement (Chen and Bargh, 1999). A future study could therefore investigate avoidance-related movements. For example, participants are requested to leave their hand rested on a dot presented on the middle, lower part of the computer screen and are asked to remove their hand as quickly as they can, once a stimulus is being presented. In addition, since angry stimuli are often related with approaching movements (see, for example Bossuyt et al., 2014), it is important to explore actions toward and away from different emotions in future studies.

In sum, the current study made use of a new device that makes it possible to directly test actions toward (or away from) emotions. Results showed a threat bias for angry faces and bodies, which is in line with previous studies showing that threat cues are prioritized over neutral cues. There was no threat bias for fearful stimuli, suggesting that the directness of the threat in case of anger sped up reaction times, rather than the negativity of a stimulus. Also, no difference was found between responding to faces and bodies, suggesting that the threat bias is general and not restricted to one source. The current study supports the notion that evolution evolution prepared humans for fast actions when facing a threat.

### Conflict of Interest Statement

The authors declare that the research was conducted in the absence of any commercial or financial relationships that could be construed as a potential conflict of interest.

## Footnotes

1 Conventionally, a test with a partial eta-squared (${\text{η}}_{\text{p}}^{\text{2}}$) higher than 0.14 and a power (1 – β) greater than 0.8 is considered to have a high effect size and is statistically powerful (Field, 2013).

2 A more liberal paired sample *t*-test without a correction for multiple comparisons yielded a statistical trend between fear and neutral, with somewhat faster response times following fear as compared to neutral *t*(32) = 1.923, *p* = 0.063.

3 A more lenient paired sample *t*-test without a correction for multiple comparisons yielded a significant difference between the two emotions, with faster reaction times following anger as compared to fear *t*(32) = 2.042, *p* = 0.049.
